# On the reliability of powder diffraction Line Profile Analysis of plastically deformed nanocrystalline systems

**DOI:** 10.1038/srep20712

**Published:** 2016-02-10

**Authors:** Luca Rebuffi, Andrea Troian, Regina Ciancio, Elvio Carlino, Amine Amimi, Alberto Leonardi, Paolo Scardi

**Affiliations:** 1 Elettra-Sincrotrone Trieste, Mechanical, Vacuum and Optic Engineering Group, Trieste, 34149, Italy; 2 Lund University, Department of Physics, Lund, SE-221 00, Sweden; 3 CNR-IOM TASC, Trieste, 34149, Italy; 4 University of Trento, Department of Civil, Environmental and Mechanical Engineering, Trento, 38123, Italy; 5 Indiana University, Department of Geological Sciences, Bloomington (IN), 47405, USA

## Abstract

An iron-molybdenum alloy powder was extensively deformed by high energy milling, so to refine the bcc iron domain size to nanometer scale (~10 nm) and introduce a strong inhomogeneous strain. Both features contribute to comparable degree to the diffraction peak profile broadening, so that size and strain contributions can be easily separated by exploiting their different dependence on the diffraction angle. To assess the reliability of Line Profile Analysis, results were compared with evidence from other techniques, including scanning and transmission electron microscopy and X-ray small angle scattering. Results confirm the extent of the size broadening effect, whereas molecular dynamics simulations provide insight into the origin of the local atomic, inhomogeneous strain, pointing out the role of dislocations, domain boundaries and interactions among crystalline domains.

Since the discovery of powder diffraction nearly a century ago, diffraction Line Profile Analysis (LPA) has been extensively used to determine size and shape of crystalline domains, and to assess presence and content of lattice defects. Even if traditional methods are nowadays described in most textbooks on powder diffraction^1^,^2^,^3^,^4^, LPA is still a subject of study^5^,^6^,^7^,^8^. Most notably, in spite of the considerable interest in LPA, there are no established procedures and no reference materials to compare results, validate methods and experimental protocols. So far Standard Reference Materials (SRMs) were only conceived to measure the Instrumental Profile Function (IPF)^9^,^10^, which is useful and functional to a proper use of LPA, but gives no support to the study of line broadening effects caused by the microstructure of materials. Recent research suggests ZnO powders as possible crystallite size SRMs^11^. The certification of the new SRM 1979, still in progress for the complexity of the microstructure, with extensive layer faulting in prismatic domains with small aspect ratio, should deliver a standard for domain size effects only.

The present paper broadens the scope to size & strain line broadening effects. In particular we report the first part of a collaborative project involving several laboratories, aimed at producing and testing a possible reference material for LPA, including both size and strain effects.

In the following we provide a characterization of the selected material, an iron-molybdenum alloy powder extensively deformed by high energy milling, and an assessment of the reliability of LPA. Recent literature only reports a Round Robin (RR) on LPA, but the aim was quite different^12^. The studied sample was a chemically synthesized nanocrystalline ceria powder, with a mean domain size of less than 20 nm. Presence of a strain broadening component was mentioned just as a minor effect, if not as an artifact of some data analysis procedures. Before this work, a similar project based on glass-ceramic specimens had been started and discussed at the first Size-Strain conference (“Size – Strain’95”, Liptovski Mikulas, Slovakia, August 21–25, 1995), but results were not conclusive, and no written report was included in the following publication^5^.

The material of the present study was selected according to several requirements, but also to be representative of a broad class of case studies in chemistry, physics and materials science. The choice of a heavily deformed bcc iron-alloy powder produced by high energy milling was supported by:

1. presence of a single phase, with high symmetry (bcc) crystal structure, such to produce powder diffraction peaks with as little overlapping as possible;
2. line broadening effects due to small domain sizes and inhomogeneous strain, with the latter caused mostly by grain boundary/grain-grain interactions and presence of a dominant lattice defect type, namely dislocations, both causing an anisotropic (*hkl*-dependent) line broadening;
3. contaminant species present in a reasonably low and controlled level, not interfering significantly with the LPA analysis, but improving stability against oxidation, thus granting a good stability in time;
4. large amount of material available by a simple, inexpensive and reproducible preparation process.

Besides providing a detailed characterization of the material, which could be the basis of a certification protocol, the present work sheds light on the mechanism of plastic deformation in metallic materials undergoing extensive grinding. Atomistic modelling based on Molecular Dynamics (MD) simulations is used to better understand the true nature of the deformed samples, and how this reflects on the observed line broadening effects. MD provides unique insights into the mechanisms responsible for the inhomogeneous strain, thus supporting an assessment of LPA meaning and reliability.

## Materials and Methods

The Fe-1.5 wt%Mo alloy (Astaloy Mo^®^, supplied by Höganäs AB^13^) is a pre-alloyed powder for large industrial production, mostly used in powder metallurgy processes. Molybdenum, entering the bcc crystal structure of iron in solid solution, is added to increase hardenability and to improve the mechanical properties by a solution-hardening effect. The latter is caused by the lattice strain introduced by alloying Mo, with a smaller atomic radius, to the host matrix of Fe. Molybdenum addition also enhances thermal stability, as it hinders grain growth (and recrystallization) processes, thus stabilizing the work-hardening effects introduced by grinding. At the same time Mo does not increase the tendency of oxidation, which would lead to unwanted secondary oxide phases^14^.

The Fe-1.5 wt%Mo (hereinafter FeMo) powder was ground in a planetary ball mill (Pulverisette 4, manufactured by Fritsch GmbH^15^), using two 250 ml jars of X210Cr12 steel designed and made at the University of Trento^16^. Milling agents were 50 tempered steel (100Cr6) spheres, with a diameter of 12 mm and a mass of 7.1 g.

Milling parameters were based on previous studies^17^: main disk speed (Ω) was 300 rpm, whereas the speed ratio (*ω*/Ω) was −1.8, with *ω* as the rotational speed of the satellite jars. The ball to powder ratio (BPR) was set to 10:1. Grinding was made in static Ar atmosphere (O_2_ < 2%) and Room Temperature (RT) conditions, with a 4 wt% ethanol (96%vol purity) added as lubricant, to avoid cold welding and sticking of the powder to the jar walls.

Eight batches were produced and individually characterized^16^, showing similar microstructural parameters. Batch 4a was selected for the present study as it gives results close to the average values for the whole production.

X-ray Diffraction (XRD) patterns were collected at the MCX beamline of the Italian synchrotron Elettra, using the standard capillary geometry and a beam energy of 15 keV *λ* = 0.0826 nm). Details on the beamline are described in recent literature^18^. Small Angle X-ray Scattering (SAXS) data were collected at the same synchrotron radiation facility, using the standard set-up described in literature^19^.

Morphological investigation of the selected batch was carried out by a ZEISS Supra 40 field-emission gun (FEG) scanning electron microscope (SEM) equipped with a Gemini column and an in-lens detector yielding increased signal to noise ratio. The microscope is also provided with an EDAX system for energy dispersive X-ray spectroscopy (EDS) studies.

HRTEM analyses were performed by using a JEOL 2010 UHR field emission gun microscope operated at 200 kV with a measured spherical aberration coefficient, Cs, of 0.47(1) mm, which enables a resolution in phase contrast at optimum defocus of 0.19 nm.

Molecular Dynamics simulations were performed using LAMMPS code (Large-scale Atomic/Molecular Massively Parallel Simulator^20^) to simulate microstrain contribution from the defects in the microstructure (e.g. grain boundary and dislocations). The system was energy minimized and then equilibrated for 0.6 ns at constant pressure (0 Pa) and temperature (300 K) up to reaching a steady state using Nose-Hoover style non-Hamiltonian time integration and the Embedded Atom Method^21^,^22^. Next, the arrangement of atomic positions in space was sampled at 2 fs time step for a 2 ps long time trajectory, and the average configuration was finally computed to cancel the dynamic contribution out of the observed distortion field^23^.

## Results and Discussion

High-energy ball-milling of a FeMo powder has been described as a three-stage process^17^: last stage, set in after extensive grinding (>32 h), yields a homogeneous microstructure made of roughly equiaxed nanocrystalline domains with a high density of dislocations. However, even under the most effective conditions, ball-milling effects tend to saturate as a dynamical equilibrium establishes between plastic deformation and annealing, so that the domain size and lattice defect content do not change any further with additional milling.

**Figure 1:**
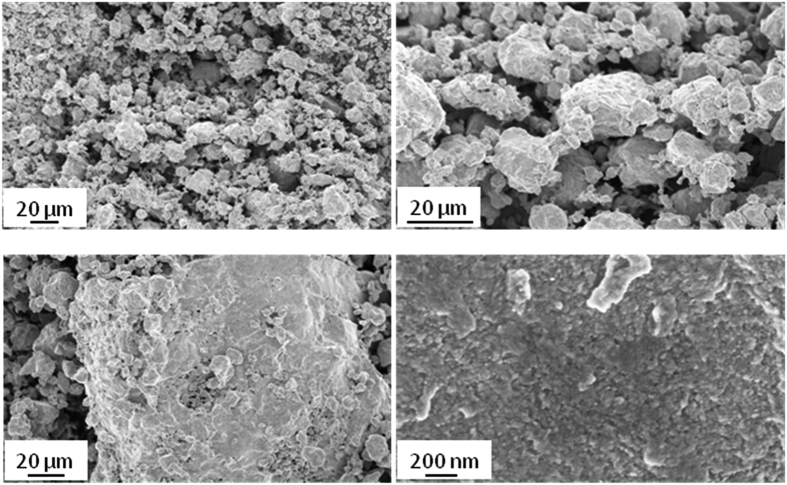
SEM micrographs at different magnification of the FeMo powder ground for 64 hours. Additional micrographs of the FeMo powder after shorter ball-milling are reported in the Supplementary Information file.

With the milling equipment used in this work, 64 hours is the appropriate grinding time for the production of a large amount of powder with a uniform microstructure^16^,^17^ (see also Supplementary Information file). The total time including testing, run in of jars and balls, and production of 4 batches is suitable to guarantee stability of the grinding media, which undergo rather severe in-service degradation. Powder particle morphology is also important. While the extensive grinding decreases the grain size to nanometer scale, as a result of a fragmentation process triggered by localized deformation, there is a strong tendency toward agglomeration. Starting from the ~90 *μ*m grain size of the pristine powder, Fig. 1 shows that ball milled particles range from a few to a several tens of micrometers.

At the highest magnification nanocrystalline regions can be outlined as fine features in much larger particles. A tentative analysis indicated a mean size of ~15 nm (standard deviation of ~4 nm). Small Angle X-ray Scattering (SAXS) of the same ball milled FeMo powder dispersed in water (see also Supplementary Information file) gives a mean size of ~16 nm (standard deviation of 8 nm), in good agreement with the finest particle size suggested by SEM. It is clearly understood this is not the size of the coherently scattering crystalline domains (aka crystallites), features shown later by LPA and TEM, but it can be considered as an upper limit to the crystallite size. It is worth noting that agglomeration shown by SEM has a positive effect on the FeMo powder for the specific purpose of this work, because it protects against oxidation the large fraction of nanocrystalline domains inside the much larger (tens of micrometers) agglomerates shown in Fig. 1.

Spontaneous passivation, in fact, only concerns the agglomerate surface, thus limiting the formation of oxide phases which indeed are not observed in the diffraction pattern, even after long time^24^. More details are in Supplementary Information file, where XRD patterns for increasing milling time also demonstrate how oxide phases disappear for long grinding time (> 32 h) as an effect of the growing Cr contamination and agglomeration of the nanocrystalline domains.

Further investigation on this important issue was made by SEM, with the support of EDS to assess compositions and level of contamination from the grinding media. The pristine powder has an average composition of 98.7%wt Fe/1.3 wt% Mo, against the nominal 98.5/1.5 ratio, and just traces of other elements. EDS of ball milled powders shows chromium and nickel contamination from vial and balls increasing with time. Interestingly, the powder is highly reactive after a short (≤16 h) milling time, when contamination is still quite low, showing pyrophoric activity if exposed to air. Such a violent oxidation does not take place after longer grinding, when agglomerates form and, sufficiently high Cr contamination is achieved. Stable powders are obtained after 64 h grinding, when Cr and Ni content in the powder stabilize to 3 wt% and 1 wt%, respectively, in good agreement with the overall chemical composition of the milling apparatus. Then the Cr-Ni contamination acts as a mechanical alloying, mostly localized on the surface of the agglomerates, with the positive effect of protecting FeMo from oxidation, granting stability in ordinary use and storage conditions of the powder. This important aspect is further discussed below.

**Figure 2:**
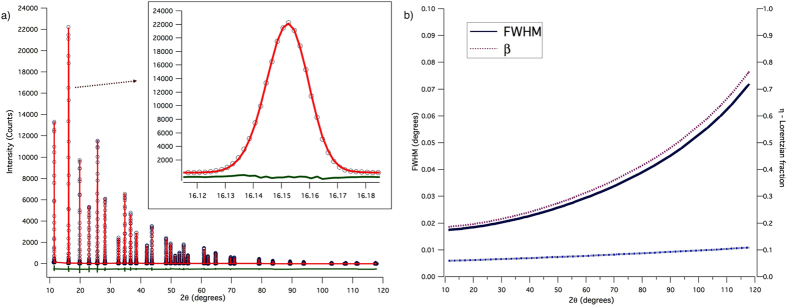
Instrumental Profile Function collected at the MCX beamline, Elettra-Sincrotrone Trieste using SRM 660a (LaB_6_): experimental pattern (circle), modelling (line) and residual (difference, line below), with a detail of a peak profile in the inset (**a**); parametrization of the IPF, with Full Width at Half Maximum (FWHM), integral breadth (*β*), and Lorentzian fraction of the pseudo-Voigt IPF line profile (*η*) as a function of the diffraction angle (**b**).

The XRD data for all powder batches, including the sample of the present study, were collected at the MCX beamline of the Italian synchrotron Elettra. As a significant advantage in LPA studies, MCX features a carefully controlled IPF, which can be effectively parametrized and incorporated in LPA algorithms^18^. Figure 2 shows the experimental pattern and modelling of the SRM 660a (LaB_6_)^9^.

Before LPA, FeMo data were corrected for absorption effects. The transmitted beam was measured under the same experimental conditions used to collect the powder diffraction data, by moving the capillary through the beam aimed straight at the detector^16^. Same procedure was repeated for the studied capillary and an empty one. A transmitted beam percentage of 9.2(1)% was measured, corresponding to a FeMo powder packing factor of 46% and a product of absorption coefficient (*μ*) and capillary radius (*R*) 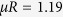. Standard expressions for capillary geometry^25^ were used to correct the powder data for absorption^16^.

The corrected FeMo data were analyzed by Whole Powder Pattern Modelling (WPPM)^7^,^26^, following a well-assessed procedure^17^. The equiaxed crystalline domains were described as a system of spheres with lognormally distributed diameters, containing straight dislocations of screw and edge type. Diffraction line profile components related to domain size/shape and to dislocations were convolved with the IPF to model the experimental data by non-linear least squares minimization^7^,^26^.

Free microstructural parameters in the refinement procedure were: lognormal mean (*μ*) and variance (*σ*) of the diameter distribution, average dislocation density (*ρ*) and effective outer cut-off radius 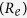. The anisotropic broadening effect of dislocations, according to the Krivoglaz-Wilkens theory^27^,^28^,^29^, was described by an average contrast factor calculated for screw and edge dislocations in the primary slip system of bcc iron 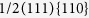
17. To account for the dislocation type, an edge/screw fraction parameter 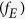 was also refined. Additional refinement parameters include the unit cell parameter 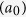, coefficients of optical aberration functions (horizontal/vertical position of the capillary) and background. Concerning the latter, the pattern from an empty capillary was modeled by a set of pseudo-Voigt functions, and this model, with all parameters fixed but a single refinable scale factor, was added to a Chebyshev polynomial to fit the background in the analysis of the FeMo data.

**Figure 3:**
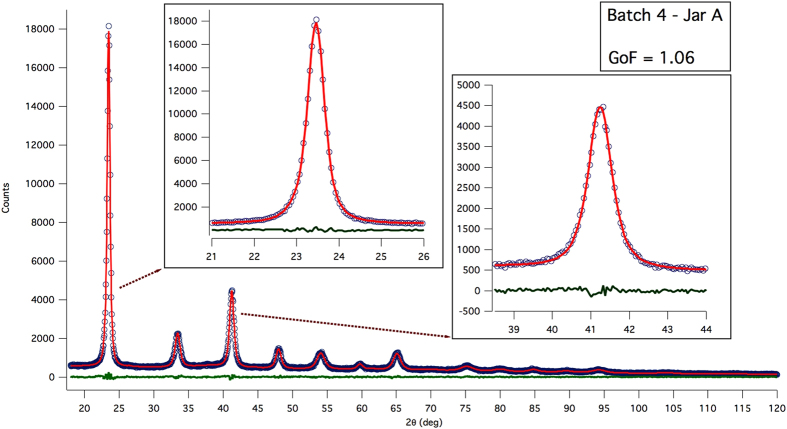
WPPM results: experimental data (circle), model (line) and residual (line below). Insets show details of the modelling of the two most intense reflections.

**Figure 4:**
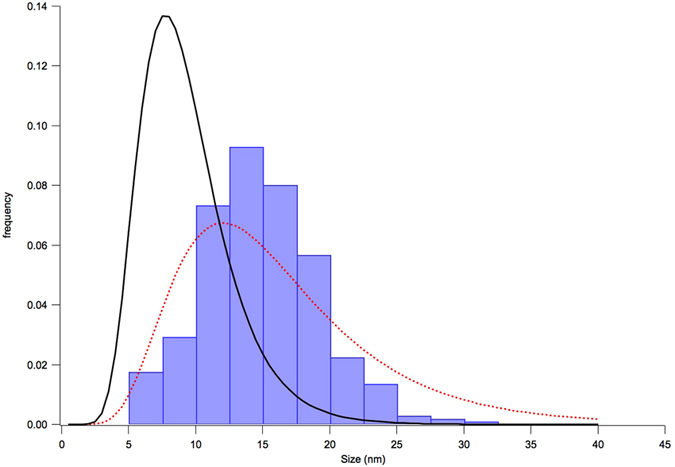
Histogram of particle size from SEM micrographs (400 particles, min. size limit 5 nm, mean 14.5 nm, s.d. 4.2 nm); lognormal size distribution of diameters from SAXS (red dot: mean 16.2 nm, s.d. 7.6 nm) and Whole Powder Pattern Modelling (WPPM) of XRD data (black line: mean 9.3 (8) nm, s.d. 5.9 (9) nm). See text for details.

Figure 3 shows the WPPM result, with details on the two most intense reflections in the insets. Modelling quality is good, even if some non-random feature is visible in the residual for the most intense (lowest angle) reflection. The corresponding distribution of spherical domain diameters is shown in Fig. 4, together with the particle size distributions from SEM (histogram) and SAXS (dot line) already discussed above: as expected, coherent domains are a finer feature than the particles seen by SEM and SAXS.

Arithmetic mean size is 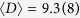 nm, with s.d. of 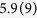 nm. It is also worth considering the volume-weighted and surface-weighted mean sizes, respectively, 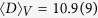 nm and 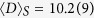 nm. The former is related to the integral breadth of the diffraction peaks (through a shape factor, aka Scherrer constant, 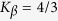
30,31), whereas the latter is usually evaluated by the Warren-Averbach method^32^,^33^ (with a corresponding shape factor of 3/2^30,31^). Owing to the relatively little dispersion (narrow domain size distribution) differences among the three mean values are correspondingly small (< 30%).

The WPPM analysis also gives parameters of the elastic strain profile component, which in terms of dislocations effect are: 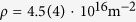, 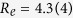 nm, 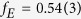. It is worth noting that the so-called Wilkens parameter 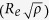 is about unity, just within the validity limits of the theory^28^,^29^, possibly suggesting a strong dislocation interaction, as in dislocation walls and dipoles^28^,^29^. While the effective outer cut-off radius is about half mean domain size, which is not an unreasonable value, questions may arise about the actual meaning of *ρ*. The dislocation density is quite high, as it would correspond to about two dislocations per spherical domain (for a mean diameter of 9.3 nm: 
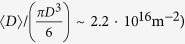
.

**Figure 5:**
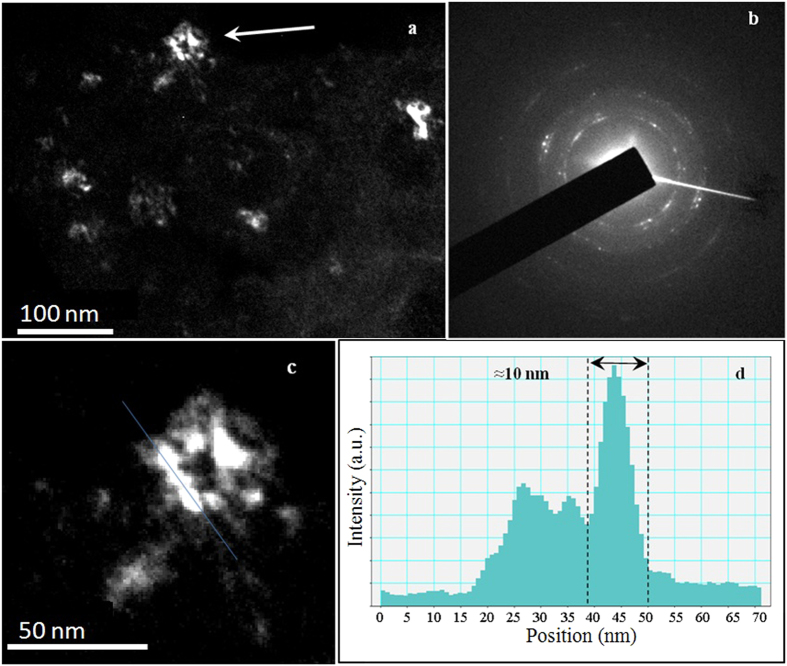
Dark field TEM pictures of a ball milled FeMo powder specimen. Overview of the microstructure (**a**) and corresponding electron diffraction pattern (**b**); detail of the region pointed by the arrow (**c**), with line scans across image intensity maxima (**d**). See text for further details.

TEM provides an indispensable support to the present study, to shed light on the actual size of the FeMo domains and on the lattice defects. TEM showed the internal fragmentation of the FeMo particles, not accessible to SEM or SAXS due to their intrinsic resolution limits. A representative overview of nanocrystalline FeMo is shown by the dark field image of Fig. 5a obtained by selecting a single (0, *k, l*) FeMo diffraction spot to form the TEM image. Overall, grains tend to cluster into equiaxed agglomerates with random orientation (cf. the electron diffraction pattern in (b)) ranging from 20 to 50 nm average size, which in turn consist of enclosed bright contrast sub-grain units. The dark field image in (c) depicts, at higher magnification, the representative case of the cluster pointed in (a), where the internal fragmentation can be distinctly appreciated.

Line scans across image intensity maxima measured along relevant portions of the imaged cluster confirmed the enclosed presence of domains of about 10 nm, in good agreement with the XRD/WPPM results.

**Figure 6:**
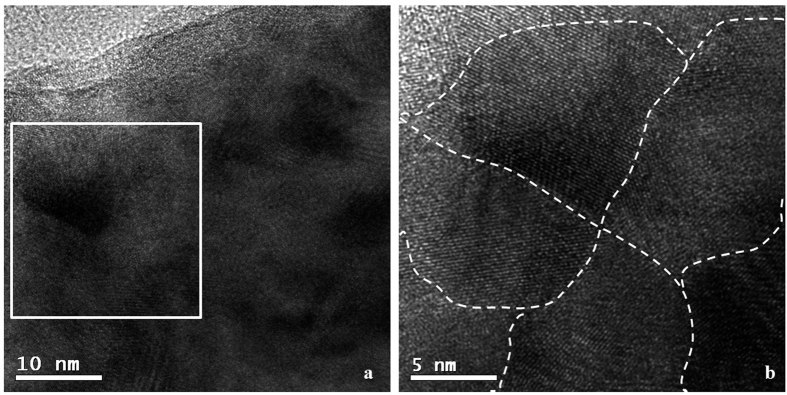
High resolution electron microscopy picture of a ball milled FeMo powder specimen. Region framed by the square in (**a**) is shown at higher magnification in (**b**), with indication of the grain boundaries (dash lines).

High Resolution Transmission Electron Microscopy (HRTEM) provides details on the high-angle grain boundary structure surrounding crystalline domains. The micrograph in Fig. 6 shows an example: the area framed by the white box in (a) and shown at higher resolution in (b) depicts the case of four grains facing to each other where the boundaries have been marked by dashed lines.

**Figure 7:**
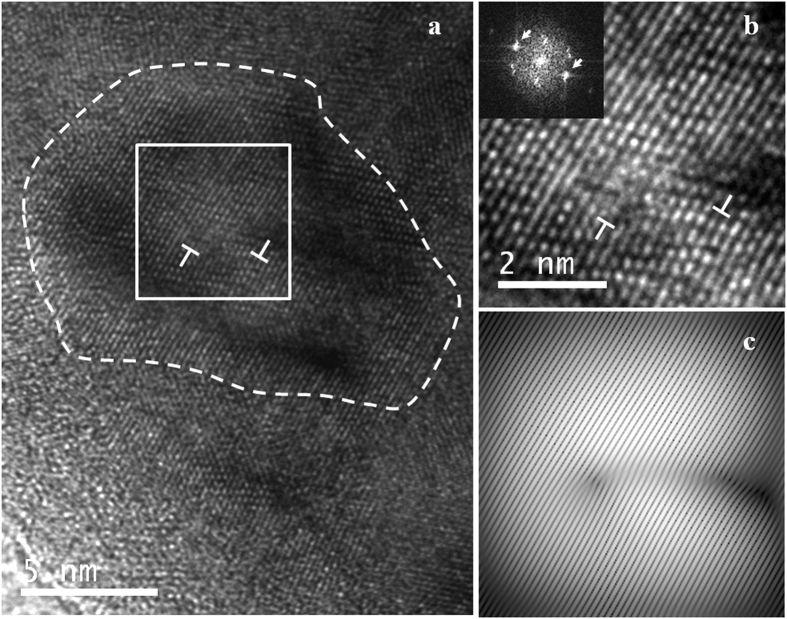
High resolution electron microscopy picture of a ball milled FeMo powder specimen, showing a grain with trapped dislocations (**a**); a higher magnification picture is shown in (**b**), with the FFT in the inset; filtered picture, as obtained by inverse FFT to highlight the dislocation, is shown in (**c**). See text for details.

Again, TEM observations point out the presence of separate domains of about 10 nm size. This further confirms the capability of WPPM/XRD for measuring the distribution of coherently scattering domains, in agreement with the specific literature^24^,^34^,^35^,^36^. The HRTEM micrograph of Fig. 7 focuses on a representative grain, marked by the dashed line, where trapped dislocations are visible (indicated by 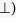. The strong ripple contrast in the image may be taken as an indication that the area was experiencing substantial strain. The dislocation area framed by the white box in (a) is shown at higher magnification in (b), where the corresponding FFT is included as an inset. The most intense diffraction spots (arrows) indexed in the FFT are compatible with those of the (110) planes of bcc FeMo, whereas weaker spots are compatible with a Fe_3_O_4_ spinel phase. Such a minority oxide phase (totally absent in the XRD patterns) only appears on the loose particles of thickness suitable to the electron transmission, mostly present on the surface of the large clusters (see Fig. 1) composing the powder. Figure 7c is the corresponding inverse FFT image of the dislocation area, reconstructed using the spatial frequencies of the (110) planes of bcc FeMo. This picture further highlights the trapped dislocations, as well as a distinct dark contrast resulting from the propagation of the strained area during the stress relief. The measured displacement vector at each dislocation site is about 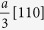, which is compatible with a Burgers vector 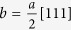 of a unit edge dislocation in the primary slip system of bcc iron. Dislocations appear in a dipole configuration, with strongly interacting strain fields, probably as a result of tendency to minimize the total energy^37^.

The TEM investigation reported so far qualitatively supports the results of the XRD/WPPM analysis. However, owing to the thickness-selectivity of HRTEM experiments, only a few grains can be analyzed across the large FeMo clusters (Fig. 1) and additionally, reliable data can only be extracted from grains properly oriented with respect to the electron beam. Therefore dislocations may be difficult to observe due to unfavourable orientation, and because of screening effects of the metal cluster where domains are embedded in. However, based on the result of extended TEM experiments, presence of dislocations is most likely restricted to a few domains.

This evidence would suggest that strain broadening effects in the XRD line profile could be just partly related to dislocations; additional elastic strain components could arise from the domain boundary, which incorporates dislocations transiting across crystalline domains during plastic deformation^38^,^39^,^40^,^41^,^42^, and by interactions among different domains (so-called grain interaction), thus resulting in a mix between type II (intergranular) and type III (intragranular) strains^43^,^44^,^45^,^46^,^47^.

**Figure 8:**
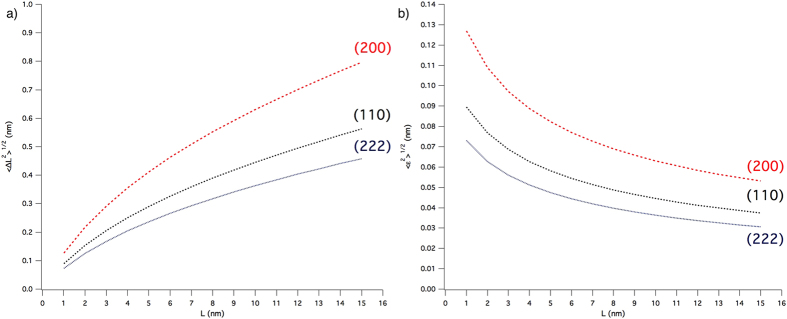
Warren’s plot for ball milled FeMo powder. r.m.s. displacement (**a**) and r.m.s. strain (**b**) along different crystallographic directions. See text and Appendix A for details.

It is therefore logical to conclude that the dislocation density obtained by means of the Krivoglaz-Wilkens theory is just an upper limit to the true value. To the purpose of using this material as a reference, and in general, to provide a more reliable result, it is convenient to refer to the r.m.s. strain (root mean square elastic strain, or microstrain, 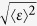, see Appendix A in Supplementary Information file), the width of the strain distribution in the material over different correlation lengths, *L*^46^. This representation of the strain broadening is more general, and does not commit to a specific source of strain; moreover, the WPPM analysis can easily provide a microstrain trend along any desired crystallographic directions, to highlight the effect of anisotropy. The microstrain for the ball-milled FeMo powder is shown in Fig. 8 along three representative directions; besides microstrain, also the r.m.s. displacement 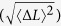 is shown, as originally proposed by Warren^32^. As expected, strain is larger along [*h*00], which is the elastically soft direction of iron. Additional advantages of reporting the strain broadening effect in terms of microstrain or r.m.s. displacement include the possibility to directly compare the present results with those provided by other methods (e.g., the Warren-Averbach method^32^,^33^); quoting a microstrain also avoids intrinsic problems with the Krivoglaz-Wilkens theory, as the simultaneous refinement of *ρ* and *R*_*e*_ can be unstable owing to the strong correlation between the two parameters^47^.

Even if of no direct or critical importance in the context of a LPA reference material, the unit cell parameter contributes some useful additional information. Measured value is 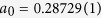 nm, well above the unit cell parameter of the pristine material, 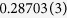 nm^7^, which in turn agrees well with the ideal solid solution value for Fe-1.5 wt%Mo (0.28706 nm, based on Vegard’s law^48^,^49^).

A larger unit cell parameter is typical of ball-milled metals^7^,^26^, mostly because of the effect of severe plastic deformation, with additional contributions by alloying (solid solution) with materials of the grinding media (Cr and Ni in this case). Very fine crystalline domains increase vacancy and defect solubility^50^, leading to volume expansion, which adds to the free volume increase caused by extrinsic dislocations piling up at the grain boundaries^38^.

It is also worth nothing again that, despite the high level of elastic energy stored in the ball milled material, the FeMo microstructure is quite stable. A recent study demonstrated that the stabilization mechanism is mostly based on the high lattice strain, which locks grain boundaries preventing any evolution of domain size and strain up to ~100 °C^51^,^52^. Recovery starts above this temperature, supported by a dislocation cross-slip and annealing mechanism, indirectly observed as a steady decrease, above 100 °C, of the screw dislocations. This activation mechanism is probably necessary because screw dislocations in iron tend to be non-gliding^53^. This evidence suggests that an extensively ball milled FeMo powder is stable under common measurement conditions and storage at RT, a rather fundamental feature for a candidate reference material. Stability of the microstructure is paralleled by the resistance to oxidation, which besides the action of Molybdenum^14^ has been shown to be granted by the Cr-Ni contamination from the grinding vial and balls. As a direct experimental validation, both features were verified in a recent study, where a ball milled FeMo powder has been measured ten years after grinding^17^, providing the same size/strain and unit cell parameters within experimental error, and no measurable signals from oxide phases^16^,^24^. This stability in time can only be improved by the present grinding conditions, which increase the Cr/Ni content with the respect to the past study (see Supplementary Information file for details).

**Figure 9:**
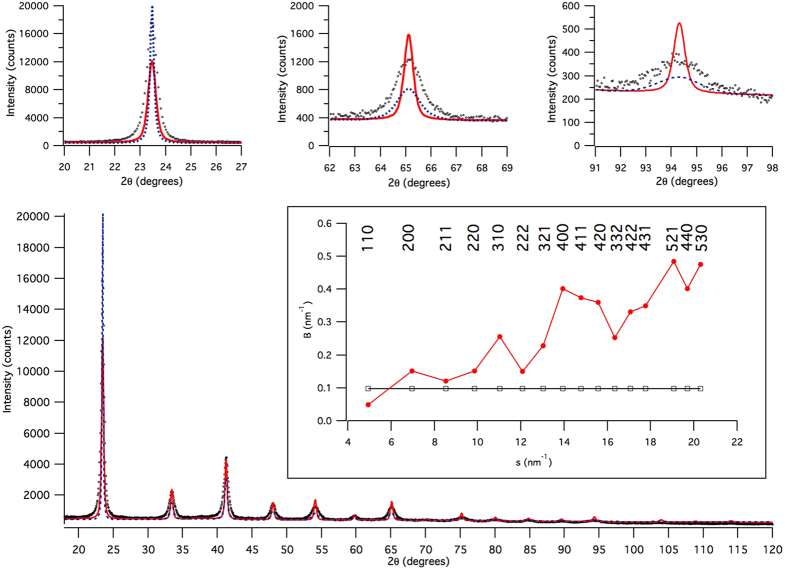
X-ray powder diffraction pattern of the FeMo powder: experimental data (circle), crystalline domain size (red line) and inhomogeneous strain (blue dash) profile components. Pictures above show details from low to high 2*θ* angle, respectively, (110), (321), and (431) peaks of *α*-iron. The total model profile is given by the convolution of the two components (cf. Fig. 3). The inset shows the integral breadths for the size (open square) and strain (full circle) components, as a function of *s*, the scattering vector modulus (aka Williamson-Hall plot).

Results shown so far provide the necessary information to use this powder as a reference material in diffraction size-strain analysis: plots of Fig. 8 may serve as a measurement of microstrain level and anisotropy, whereas the domain size, shown by TEM pictures for individual domains, is quantified by the distribution of Fig. 4 provided by the WPPM analysis. It is also worth noting that size and strain effects contribute to a comparable extent to the profile broadening. This feature is shown in Fig. 9 and insets, where the line profile components of the size (line) and strain (dot) effects are shown with the experimental data. While at low angle the size profile component is broader that the strain profile component, the opposite is true for increasing 2*θ* angle, with the trend of the integral breadth (IB, ratio between peak area and peak height) shown in the inset for the two profile components. While the IB of the size profile (open symbol) is constant as a function of the scattering vector modulus, the IB of the strain profile (full symbol) increases with a characteristic dispersion about a linear trend, caused by the anisotropy of the elastic medium and of the dislocation strain field. This feature, combines with the other useful properties shown so far – including stability in time and low contamination effects, availability by a simple and reproducible process, presence of a single phase, and intense diffraction signal with limited peak profile overlapping – making the ball milled FeMo alloy powder an ideal candidate for a LPA reference material.

### Atomistic modelling of Fe nanocrystalline domains with dislocations

Despite the clear separation of the size and strain line profile effects, a better understanding of the origin of the microstrain is still required. As shown in the previous paragraph, if strain broadening is entirely attributed to dislocations according to Wilkens model, the WPPM analysis gives two dislocations per domain, which seems rather unrealistic according to most literature and our own TEM evidence. In fact, it is known that the number of visible dislocations in extensively deformed metallic materials, after grinding crystalline domains down to a nanostructure, is not so large: dislocations generated during the deformation process transit across crystalline regions and in most cases merge into the grain boundary^39^,^40^,^41^,^42^,^54^.

To understand the possible role of dislocations in our ball-milled sample we can test the WPPM analysis under the restrictive hypothesis of an effective outer cut-off radius extended to the entire crystalline domain, 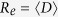, as if there were a dislocation in the centre of each grain^28^,^47^. Even with this constraint, data modelling gives 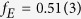 and 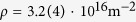, which is still too high a value to be realistic.

Further evidence is provided by atomistic modelling. An aggregate of 50 Fe grains (Fe50g) was created by means of a modified Voronoi tessellation algorithm^55^,^56^, based on the information provided by WPPM analysis and TEM pictures. The tessellation procedure, which can be driven to obtain a given distribution and a desired deviation from an equiaxial grain shape, was made according to the domain shapes of the TEM pictures, with sizes in agreement with the WPPM distribution of Fig. 4.

After filling with Fe atoms the system was allowed to equilibrate, minimizing energy and performing the dynamics according to the EAM pair potential. The MD was run until a steady state was reached (usually 0.5 ns), considering the following cases: absence of any defects other than grain boundaries (free case); presence of one edge dislocation in a selected grain, G5, whose size, in terms of diameter of a sphere with same volume, is equivalent to the mean size refined by WPPM, 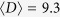 nm (1edge case); same conditions, but the dislocation in G5 was unstable and slid out during the MD trajectory, merging into the grain boundary region (1edge unstable case).

**Figure 10:**
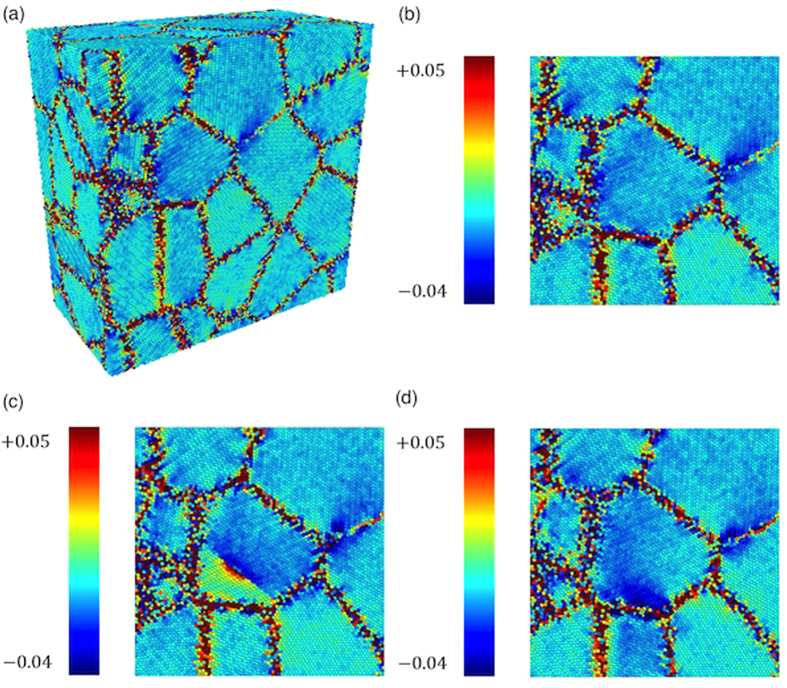
Cross-section of the cluster of 50 Fe grains (**a**), after Molecular Dynamics with detail of grain 5 (G5) free of defects other than the grain boundary (**b**), G5 with one edge dislocation and (**c**), and G5 with an unstable dislocation, merged into the grain boundary (**d**). Colour scale refers to the volumetric strain, as obtained by the VCD method (see text for details).

Figure 10 shows a cross section of the Fe50g cluster (a), with a close-up view of grain G5 in free (b), 1edge (c) and 1edge unstable (d) conditions. The colour scale, according to the Voronoi Cell Deformation (VCD) method^55^, shows the volumetric strain. Strain is high in the grain boundary region, but is also quite strong in the 1 edge case, where the picture shows the compression/tension regions introduced by the edge dislocation (lying perpendicular to the drawing plane), which makes the system quite unstable. The excess energy easily drives dislocation glide to the grain boundary, so that the 1edge unstable case (d) was the most frequently observed case in the MD simulations with dislocation containing G5. The 1 edge case in (c), with the line defect still present after MD, was only obtained after several attempts, randomly positioning and orienting the dislocation line in different ways at each new MD trajectory, and avoiding any overheating process even for short time period. This supports the conclusion that edge dislocations are unstable inside small iron domains^41^,^42^,^54^,^57^,^58^.

The atomic coordinates of G5 were used in the Debye Scattering Equation (DSE) to generate powder patterns^59^. For each different case (free, 1edge, 1edge unstable), patterns obtained at 100 different time steps in the final part of the MD trajectory were averaged together to give a realistic simulation of an “experimental” powder diffraction pattern collected at room temperature. As a reference, a powder pattern was also calculated from the starting atomic coordinates of G5 (crystallographic case), before MD. All these patterns were then analyzed by WPPM to compare results with those from the experimental pattern.

**Figure 11:**
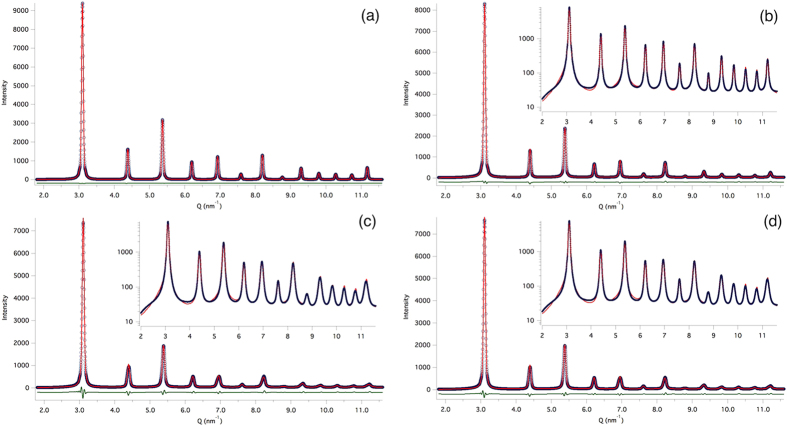
Powder diffraction patterns (circle) from atomistic simulations of grain G5 in the Fe50g cluster of Fig. 9: crystallographic, i.e., before MD (**a**); and after MD in free (**b**), 1edge (**c**), and 1edge unstable (**d**) cases. The line is the WPPM result; residual is the line below. Insets show corresponding log-scale plots.

The Common Volume Function (CVF) of G5, calculated according to Leonardi *et al*.^60^, provides a nearly perfect model for the domain size profile component in the G5 pattern. This is shown in Fig. 11a for the crystallographic case, where the only source of line broadening is the finite size and shape of G5.

The modelling of the size component in free case was made using the same CVF as in the crystallographic case, but allowing the refinement of a re-scaling parameter (99% of pristine volume) to account for the volume contraction caused by the equilibration on starting the MD trajectory. Microstrain due to grain boundary and grain-grain interactions was treated by a recently proposed model (see Appendix A), which adapts to different inhomogeneous strain sources and includes anisotropy^34^,^47^. The result, shown in Fig. 11b, is satisfactory. The modelling of the powder patterns for the 1edge case (Fig. 11c) was made using the rescaled CVF for the size effect and two microstrain sources: the grain boundary/grain-grain interaction, refined at the same time on the data of the free case, and the Wilkens-Krivoglaz model for dislocation line broadening. This corresponds to combine quadratically the microstrain given by the two effects, as also shown in Appendix A. As there is a single dislocation in G5, we can constrain the effective outer cut-off values to 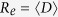. A similar approach was used for the 1edge unstable case. Modelling quality in these two cases, G5 with 1 edge or 1 edge unstable, is not as good as for the geometrical or free cases, but enough to capture the main components of line broadening.

**Figure 12:**
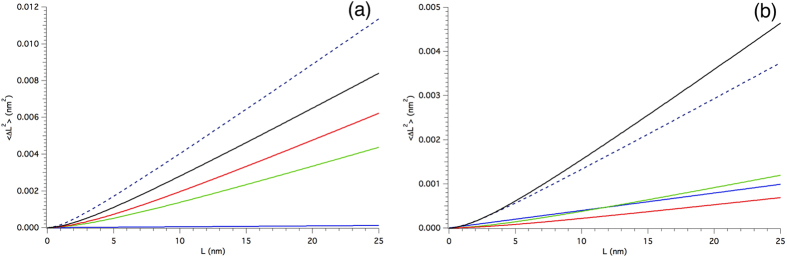
Mean square displacement vs correlation length (〈Δ*L*^2^〉 vs. *L*) along the elastically soft [*h*00] (**a**) and stiff [*hhh*] (**b**) directions. Results of the WPPM analysis of the experimental data (dash) and different simulations of the powder pattern of grain G5: free (blue), 1edge (red), 1edge unstable (green), and dipole (black).

Figure 12 summarizes some results of the modelling of simulated G5 patterns, together with the experimental results from Fig. 8. Since the grain boundary/grain-grain model and the dislocation model are combined quadratically, the mean square displacements 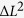 is shown for the two (elastically) extreme directions, [*h*00] and [*hhh*].

Simulations give a mean squared displacement well below the experimental results, showing again that even 1 dislocation per grain would not match the observed strain broadening. It is also worth noting that the values of the unstable dislocation case are just ~30% lower than those for the stable case, compatibly with the fact that even if dislocations glide away into the grain boundary they still contribute to the strain field considerably. In both cases, as a further confirmation of the plausibility of the results, the unit cell parameter was larger than in the corresponding free case, a result also in agreement with the experimentally observed increase of unit cell parameter.

Since we have TEM evidence for the presence of dipoles, we also tested this kind of defect in G5, but the dipole was always unstable and moved to grain boundary in a few steps of the MD trajectory. The dipole could only be stabilized during the MD if generated by adding half-planes (instead of removing them). This shows once again the large instability of line defects in nanocrystalline iron domains. However, the result in terms of mean square displacement from the WPPM analysis of the simulated powder pattern of G5 with a dipole is also shown in Fig. 12, and gives values not far from the experimentally observed level of mean square displacement.

From the simulations and modelling we can therefore conclude that line defects, if present in each grain would justify ~50% of the observed microstrain for single edge dislocations, and about 100% for dipoles. However, the clear suggestion of the literature and our own TEM evidence suggest that their number is much smaller, as many domains appear completely free of such defects. We have also observed how dislocations, even if they glide into the grain boundary, still contribute to the inhomogeneous strain; they also contribute an overall increase of average unit cell parameter caused by the introduction of free volume^38^. Even if our MD simulations are limited to very short times, and cannot account for diffusion-driven processes, and in general for all what happens on a longer time scale (e.g., annealing effects), it is plausible to conclude that the observed microstrain is only partly caused by line defects (single dislocations or complex defects as the dipole shown in Fig. 7) lying inside the crystalline domains. A major role is played by the grain boundary, which accumulates excess volume from dislocations gliding into this interface region. Further studies will be required to understand these features. The proposed reference materials, therefore, can serve as a benchmark also for developing modelling and new theories of the effect of plasticity.

## Conclusions

High energy grinding of an iron-molybdenum alloy powder provides a possible candidate as reference material for diffraction Line Profile Analysis. Size and strain effects contribute to a comparable extent to the profile broadening, an ideal condition to separate the two effects, and this feature combines with several useful properties, including: stability in time and low contamination effects, availability by a simple and reproducible process, presence of a single phase, intense diffraction signal with limited peak profile overlapping. As shown by SEM, small crystallites (~10 nm) are lumped together in much larger (tens of microns) aggregates. TEM evidence confirms the extent of the size broadening effect measured by LPA, arising from a system of equiaxed crystallites (i.e., crystalline domains which can be approximated by a distribution of spherical domains) with little dispersion of values around the mean diameter.

LPA also provides a microstrain value which could be read in terms of strain field from a high density of dislocations 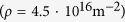 with short effective cut-off radius 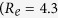 nm), equally divided in screw and edge types. Even if the result is within the validity limit of the Krivoglaz-Wilkens theory for the case of strongly interacting dislocations 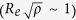, such a high dislocation density would involve the presence of quite many dislocations, about two per crystalline domain. HRTEM pictures actually show dislocations in the primary slip system of bcc iron, but they are unlikely to be present in each crystalline domains as suggested by LPA. Observed domains, even if frequently appear free of line defects, are surrounded by a complex network of domain boundaries. It is therefore possible that the high inhomogeneous strain stems from more factors, which besides dislocations involve the domain boundary and the interaction among different domains. This model, frequently envisaged in plastically deformed materials, is referred to as type II (intergranular) plus type III (intragranular) strain.

Molecular Dynamics simulations of a cluster of Fe grains similar to the experimentally observed ones support these conclusions: dislocations, and even more so dipoles, tend to be unstable in the small nanocrystalline iron domains. Even distributing one dislocation per domain cannot explain the observed microstrain level, but MD shows also how dislocations gliding into the domain boundary still contribute to the microstrain. Moreover, line defects inside domains or in the boundary cause an increase in the average unit cell parameter, as an effect of the added free volume, which is also compatible with the experimental observation.

Whatever the strain sources and their relative importance, a description in terms of r.m.s. strain as a function of *L*, the Fourier or correlation length, seems the most appropriate one to a reference material as it: (i) does not commit to any specific strain model, (ii) avoids the instability of Wilkens model, caused by the strong correlation between *ρ* and 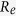, (iii) allows a direct comparison with other Fourier models, (iv) also representing the strain field anisotropy, as the r.m.s. can be reported along different crystallographic directions.

## Additional Information

**How to cite this article**: Rebuffi, L. *et al*. On the reliability of powder diffraction Line Profile Analysis of plastically deformed nanocrystalline systems. *Sci. Rep.* **6**, 20712; doi: 10.1038/srep20712 (2016).

## Supplementary Material

Supplementary Information

## References

[b1] Klug H. P. & Alexander L. E. X-ray Diffraction Procedures 2nd edn (New York: John Wiley, 1974).

[b2] Warren B. E. X-ray Diffraction. 251–314 (New York: Addison-Wesley, 1969).

[b3] Schwartz L. H. & Cohen J. B. Diffraction from Materials (Berlin: Springer, 1987).

[b4] Guinebreti`ere R. X-ray Diffraction by Polycrystalline Materials (London: ISTE, 2007).

[b5] Snyder R. L., Fiala J. & Bunge H.-J. Defect and Microstructure Analysis by Diffraction (Oxford: Oxford University Press, 2000).

[b6] Mittemeijer E. J. & Scardi P. Diffraction Analysis of the Microstructure of Materials (Weiheim: Springer, 2004).

[b7] Scardi P. Powder Diffraction: Theory and Practice. chap. 14, 376–413 (Cambridge: The Royal Society of Chemistry, 2008).

[b8] Mittemeijer E. J. Welzel E. & U. Diffraction Analysis of the Microstructure of Materials (Berlin: Wiley-VCH Verlag, 2013).

[b9] NIST. Srm 660a; lanthanum hexaboride powder line position and line shape standard for powder diffraction. Certificate, National Institute of Standards and Technology, U.S. Department of Commerce: Gaithersburg, MD, USA (2000).

[b10] NIST. Srm 660b; line position and line shape standard for powder diffraction. Certificate, National Institute of Standards and Technology, U.S. Department of Commerce: Gaithersburg, MD, USA (2010).

[b11] Cline J. P. . Crystalline domain size and faulting in the new nist srm 1979 zinc oxide. Powder Diffr. 28, S22–S32 (2013). doi: 10.1017/S0885715613001188.

[b12] Balzar D. . Size–strain line-broadening analysis of the ceria round-robin sample. J. Appl. Crystallogr. 37, 911–924 (2004). doi: 10.1107/S0021889804022551.

[b13] Höganäs, AB, Bruksgatan 35, 263 39 Höganäs, Sweden. Web site: http://www.hoganas.com (date of access: 13/11/2015).

[b14] Brenner S. Oxidation of Iron-Molybdenum and Nickel-Molybdenum Alloys. J. Electrochem. Soc. 102, 7–15 (1955). doi: 10.1149/1.2429990.

[b15] Fritsch GmbH, Industriestrasse 8, 55743 Idar-Oberstein, Germany. Web site: http://www.fritsch-milling.com (date of access: 13/11/2015).

[b16] Troian A. *Production and characterization of a microstructure reference material for X-ray powder diffraction*. Master’s thesis, University of Trento (2013).

[b17] D’Incau M., Leoni M. & Scardi P. High-energy grinding of femo powders. J. Mater. Res. 22, 1744–1753 (2007). doi: 10.1557/JMR.2007.0224.

[b18] Rebuffi L., Plaisier J. R., Abdellatief M., Lausi A. & Scardi P. Mcx: a synchrotron radiation beamline for x-ray diffraction line profile analysis. Z. Anorg. Allg. Chem. 640, 3100–3106 (2014). doi: 10.1002/zaac.201400163.

[b19] Amenitsch H. . First performance assessment of the small-angle X-ray scattering beamline at ELETTRA. J. Syn- chrotron Radiat. 5, 506–508 (1998). doi: 10.1107/S090904959800137X. 15263560

[b20] Plimpton S. Fast parallel algorithms for short-range molecular dynamics. J. Comput. Phys. 117, 1–19 (1995). doi: 10.1006/jcph.1995.1039.

[b21] Daw M. S. & Baskes M. I. Embedded-atom method: Derivation and application to impurities, surfaces, and other defects in metals. Phys. Rev. B 29, 6443–6453 (1984). doi: 10.1103/PhysRevB.29.6443.

[b22] Mendelev M. I. . Development of new interatomic potentials appropriate for crystalline and liquid iron. Philos. Mag. 83, 3977–3994 (2003). doi: 10.1080/14786430310001613264.

[b23] Leonardi A., Leoni M., Li M. & Scardi P. Strain in atomistic models of nanocrystalline clusters. J. Nanosci. Nanotech- nol. 12, 8546–8553 (2012). 10.1166/jnn.2012.6807 23421242

[b24] Troian A., Rebuffi L., Leoni M. & Scardi P. Toward a reference material for line profile analysis. Powder Diffr. 30, S47–S51 (2015). doi: 10.1017/S0885715614001298.

[b25] Maslen E. N. International Tables for Crystallography. vol. C, chap. 6.3, 599–608 (Berlin: Springer, 2006).

[b26] Scardi P. & Leoni M. Whole powder pattern modelling. Acta Crystallogr. Sect. A 58, 190–200 (2002). doi: 10.1107/S0108767301021298. 11832590

[b27] Krivoglaz M. A. & Ryaboshapka K. P. Theory of X-ray scattering by crystals containing dislocations, screw and edge dislocations randomly distributed throughout the crystal. Fiz. Metallov. Metalloved. 15, 18–31 (1963).

[b28] Wilkens M. Fundamental Aspects of Dislocation Theory. vol. II, 1195–1221 (Washington, DC: National Bureau of Standards, 1970).

[b29] Wilkens M. The determination of density and distribution of dislocations in deformed single crystals from broadened x-ray diffraction profiles. Phys. Status Solidi A 2, 359–370 (1970). doi: 10.1002/pssa.19700020224.

[b30] Langford J. I. & Wilson A. J. C. Scherrer after sixty years: A survey and some new results in the determination of crystallite size. J. Appl. Crystallogr. 11, 102–113 (1978). doi: 10.1107/S0021889878012844.

[b31] Scardi P. & Leoni M. Diffraction line profiles from polydisperse crystalline systems. Acta Crystallogr. Sect. A 57, 604–613 (2001). doi: 10.1107/S0108767301008881. 11526309

[b32] Warren B. E. & Averbach B. L. The effect of cold-work distortion on x-ray patterns. J. Appl. Phys. 21, 595–599 (1950). doi: 10.1063/1.1699713.

[b33] Warren B. E. & Averbach B. L. The separation of stacking fault broadening in cold-worked metals. J. Appl. Phys. 23, 1059–1059 (1952). doi: 10.1063/1.1702352.

[b34] Scardi P. . Anisotropic atom displacement in pd nanocubes resolved by molecular dynamics simulations supported by x-ray diffraction imaging. Phys. Rev. B 91, 155414 (2015). doi: 10.1103/PhysRevB.91.155414.

[b35] Scardi P., Leoni M., Lamas D. G. & Cabanillas E. Grain size distribution of nanocrystalline systems. Powder Diffr. 20, 353–358 (2005). doi: 10.1154/1.2135309.

[b36] Scardi P. & Leoni M. Line profile analysis: pattern modelling versus profile fitting. J. Appl. Crystallogr. 39, 24–31 (2006). doi: 10.1107/S0021889805032978.

[b37] Hull D. & Bacon D. J. Introduction to Dislocations 5th edn (Butterworth-Heinemann, 2007).

[b38] Nazarov A., Romanov A. & Valiev R. Random disclination ensembles in ultrafine-grained materials produced by severe plastic deformation. Scr. Mater. 34, 729–734 (1996). doi: 10.1016/1359-6462(95)00573-0.

[b39] Derlet P. M., Van Petegem S. & Van Swygenhoven H. Calculation of x-ray spectra for nanocrystalline materials. Phys. Rev. B 71, 024114 (2005). doi: 10.1103/PhysRevB.71.024114.

[b40] Schiøtz J. & Jacobsen K. A maximum in the strength of nanocrystalline copper. Science 301, 1357–1359 (2003). doi: 10.1126/science.1086636. 12958354

[b41] Van Swygenhoven H. & Weertman J. R. Grain boundaries and dislocations. Science 296, 66–67 (2002). doi: 10.1126/science.1071040. 11935012

[b42] Van Swygenhoven H. & Weertman J. R. Deformation in nanocrystalline metals. Mater. Today 9, 24–31 (2006). doi: 10.1016/S1369-7021(06)71494-8.

[b43] Fitzpatrick M. E. & Lodini A. Analysis of Residual Stress by Diffraction using Neutron and Synchrotron Radiation. chap. 3 and 5 (New York: Taylor & Francis Inc, 2003).

[b44] Schulze V., Voehringer O. & Macherauch E. Quenching Theory and Technology 2nd edn (CRC Press, 2010).

[b45] Skrzypek J. J., Ganczarski A. W., Rustichelli F. & Egner H. Advanced materials and structures for Extreme Operating Conditions. 178 (Berlin: Springer, 2008).

[b46] Leonardi A., Leoni M. & Scardi P. Directional pair distribution function for diffraction line profile analysis of atomistic models. J. Appl. Crystallogr. 46, 63–75 (2013). doi: 10.1107/S0021889812050601. 23396818 PMC3547226

[b47] Leonardi A. & Scardi P. Dislocation effects on the diffraction line profiles from nanocrystalline domains. Metall. Mater. Trans. A 1–11 (2015). Published online. doi: 10.1007/s11661-015-2863-y.

[b48] Vegard L. Die konstitution der mischkristalle und die raumf¨ullung der atome. Z. Phys. 5, 17–26 (1921). doi: 10.1007/BF01349680.

[b49] Denton A. R. & Ashcroft N. W. Vegard’s law. Phys. Rev. A 43, 3161–3164 (1991). doi: 10.1103/PhysRevA.43.3161. 9905387

[b50] Liu X. D., Zhang H. Y., Lu K. & Hu Z. Q. The lattice expansion in nanometre-sized ni polycrystals. J. Phys. Condens. Matter 6, L497 (1994). doi: 10.1088/0953-8984/6/34/001.

[b51] Molinari A., Libardi S., Leoni M. & Scardi P. Role of lattice strain on thermal stability of a nanocrystalline femo alloy. Acta Mater. 58, 963–966 (2010). doi: 10.1016/j.actamat.2009.10.012.

[b52] Leoni M., Scardi P., D’incau M. & Luciani G. Annealing behavior of a nanostructured fe1.5mo alloy. Metall. Mater. Trans. A 43, 1522–1527 (2012). doi: 10.1007/s11661-011-0762-4.

[b53] Duesbery M. & Vitek V. Plastic anisotropy in b.c.c. transition metals. Acta Mater. 46, 1481–1492 (1998). doi: 0.1016/S1359-6454(97)00367-4.

[b54] Ramesh K. T. Nanomaterials. Mechanics and mechanisms (New York: Springer, 2009).

[b55] Leonardi A., Scardi P. & Leoni M. Realistic nano-polycrystalline microstructures: beyond the classical voronoi tessel- lation. Philos. Mag. 92, 986–1005 (2012). doi: 10.1080/14786435.2011.637984.

[b56] Leonardi A., Leoni M. & Scardi P. Atomistic modelling of polycrystalline microstructures: An evolutional approach to overcome topological restrictions. Comput. Mater. Sci. 67, 238–242 (2013). doi: 10.1016/j.commatsci.2012.09.013.

[b57] Queyreau S., Marian J., Gilbert M. R. & Wirth B. D. Edge dislocation mobilities in bcc fe obtained by molecular dynamics. Phys. Rev. B 84, 064106 (2011). doi: 10.1103/PhysRevB.84.064106.

[b58] Chang J., Cai W., Bulatov V. V. & Yip S. Molecular dynamics simulations of motion of edge and screw dislocations in a metal. Comput. Mater. Sci. 23, 111–115 (2002). doi: 10.1016/S0927-0256(01)00221-X.

[b59] Debye P. Zerstreuung von röntgenstrahlen. Ann. Phys. (Berlin) 351, 809–823 (1915). doi: 10.1002/andp.19153510606.

[b60] Leonardi A., Leoni M., Siboni S. & Scardi P. Common volume functions and diffraction line profiles of polyhedral domains. J. Appl. Crystallogr. 45, 1162–1172 (2012). doi: 10.1107/S0021889812039283.

