# Supplementary material for: On the reliability of powder diffraction Line Profile Analysis of plastically deformed nanocrystalline systems
Source: Sci Rep. 2016 Feb 10;6:20712. doi: 10.1038/srep20712 (PMC4748273; doi:10.1038/srep20712)
Supplement: Supplementary Information [file srep20712-s1.pdf]

On the reliability of powder diffraction Line Profile Analysis of plastically deformed nanocrystalline systems

Luca Rebuffi, Andrea Troian, Regina Ciancio, Elvio Carlino, Amine Amimi, Alberto Leonardi and Paolo Scardi

SUPPLEMENTARY INFORMATION

1. SEM micrographs of FeMo alloy with progressive grinding time

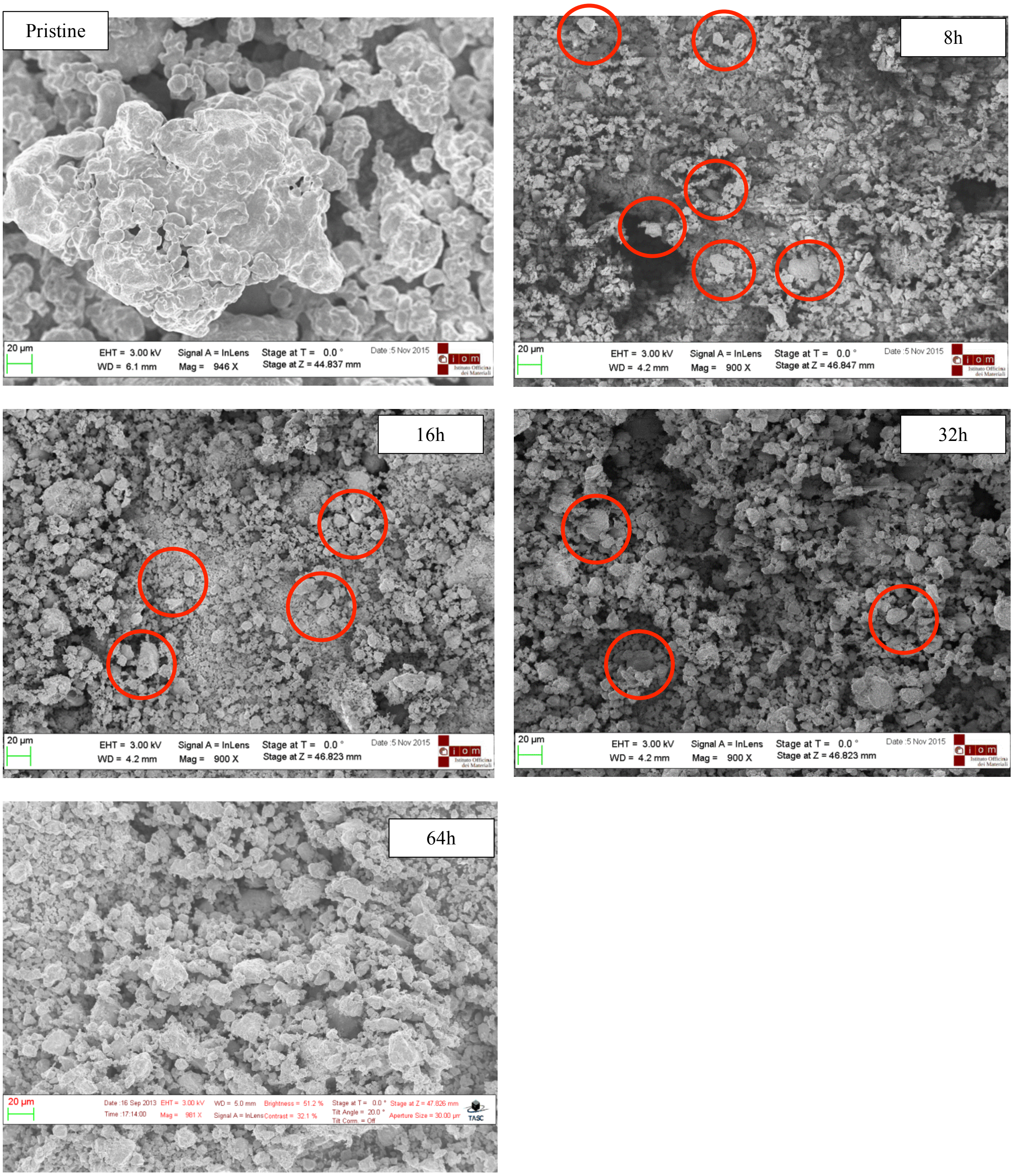

Figure SI1. SEM micrographs of the FeMo powder for increasing ball-milling time, from pristine powder to 64h (the sample studied in the present work). Even a short milling time gives extensive fragmentation of the initially large grains of the pristine FeMo powder: after 8/16h most grains are finely dispersed, although several grains are unmilled or little milled (examples are circled). A homogeneous powder can only be obtained for >32h grinding, when milled grains appear as agglomerates, from a few to a few tens of microns, made of much finer particles.

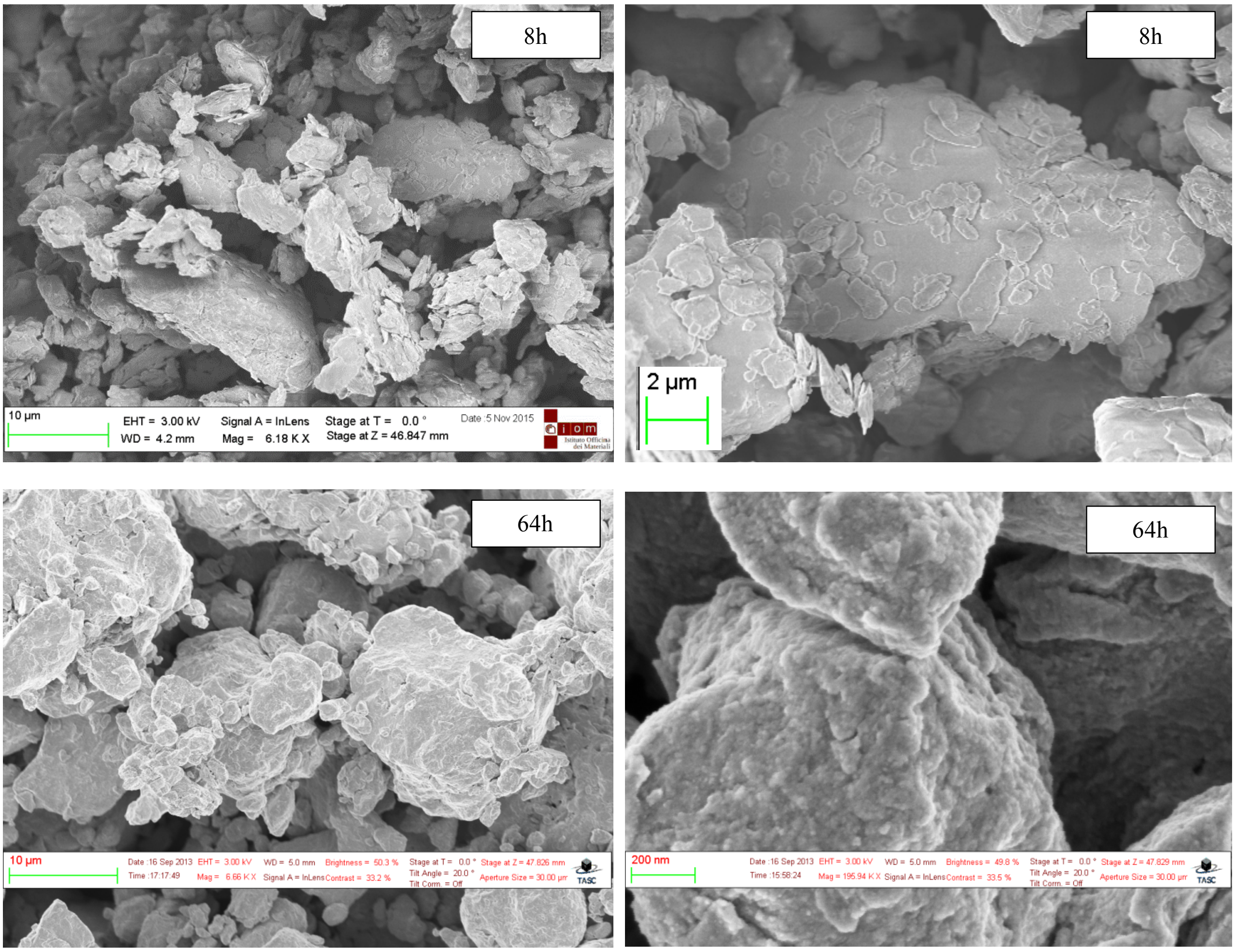

Figure SI2. SEM micrographs of the FeMo powder after 8h and 64h at higher magnification than Figure SI1. After 8h grinding plasticity effects are quite evident, and most particles appear as flattened grains; after 64h most of the material is in the form of relatively large agglomerates, which are made of much finer (tens of nm) particles. Details of the nanometre-scale crystalline domains are provided by the TEM pictures and by the Line Profile Analysis of the powder diffraction pattern shown in the main article.

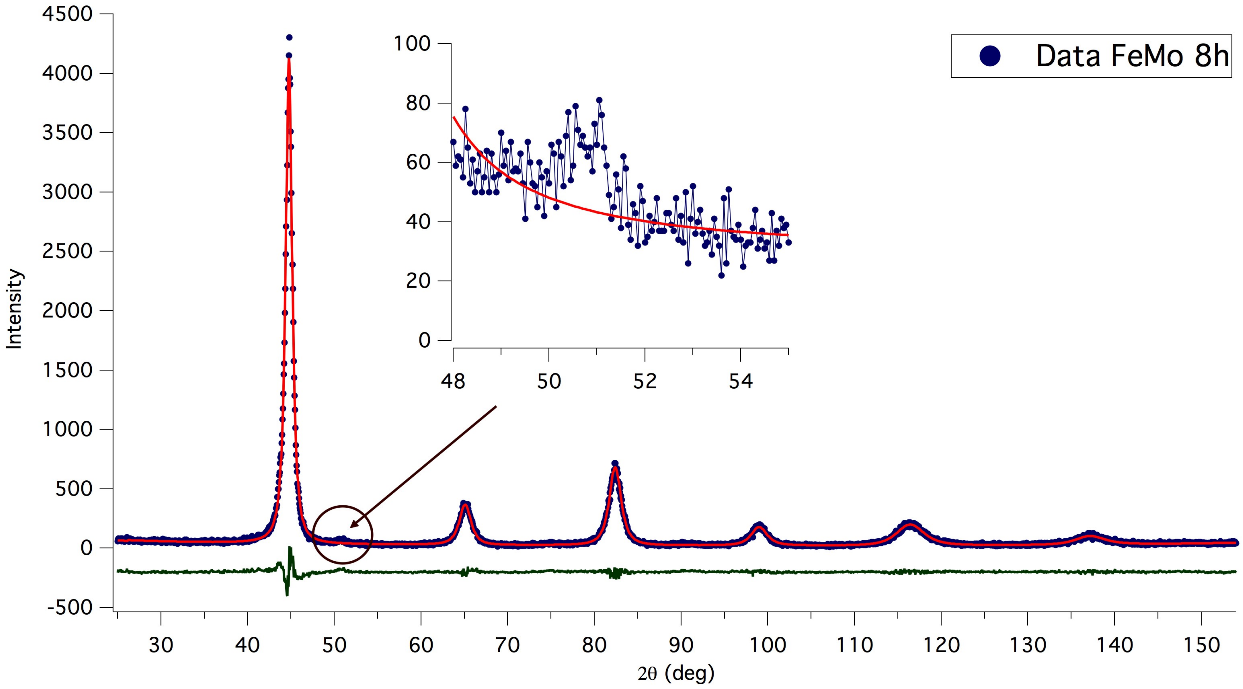
Data FeMo 8h
Intensity
2θ (deg)

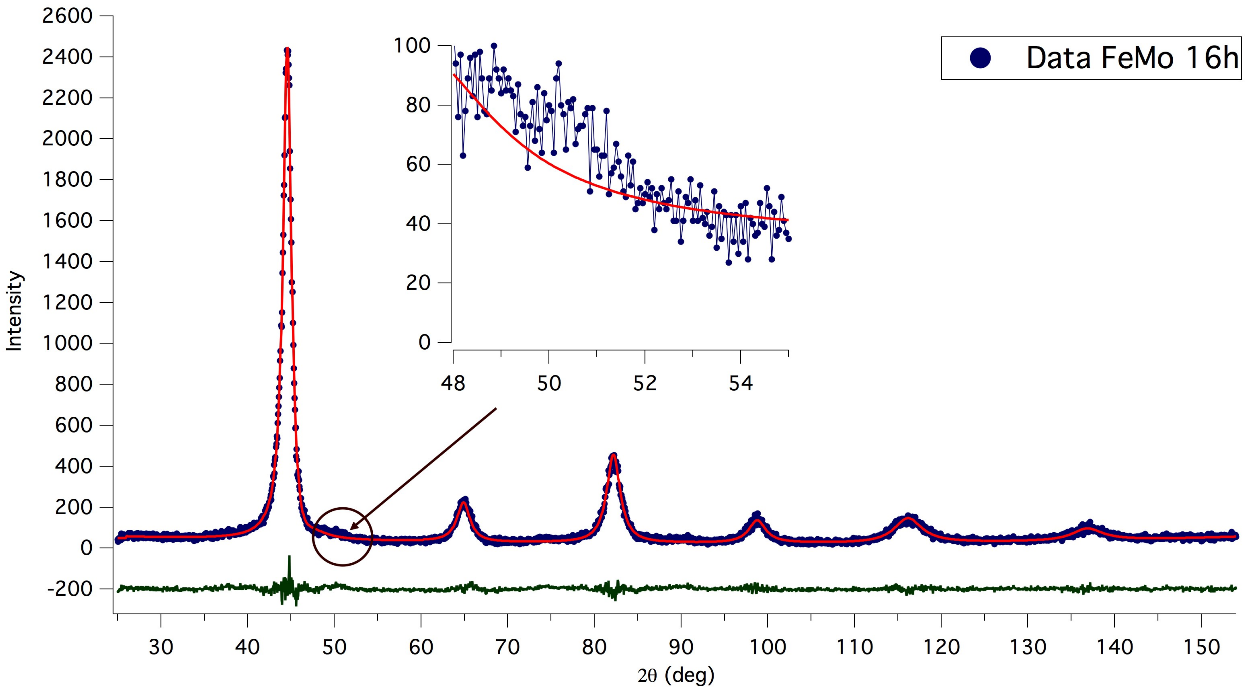
Data FeMo 16h
Intensity
2θ (deg)

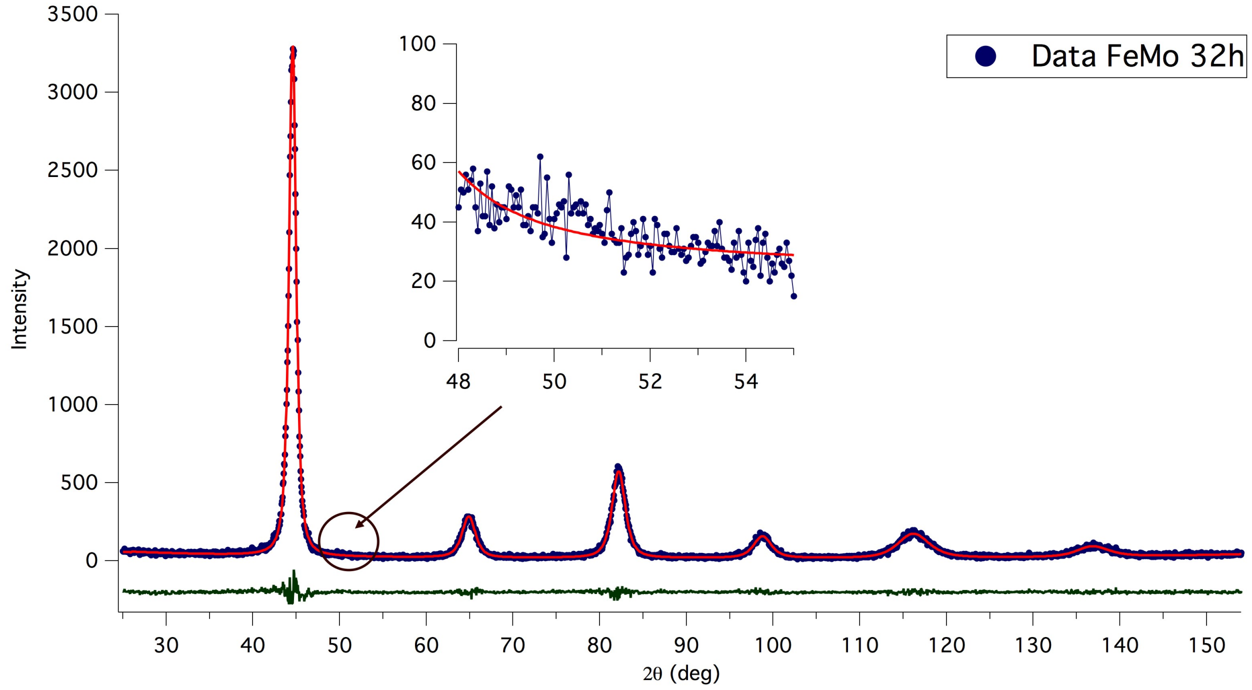

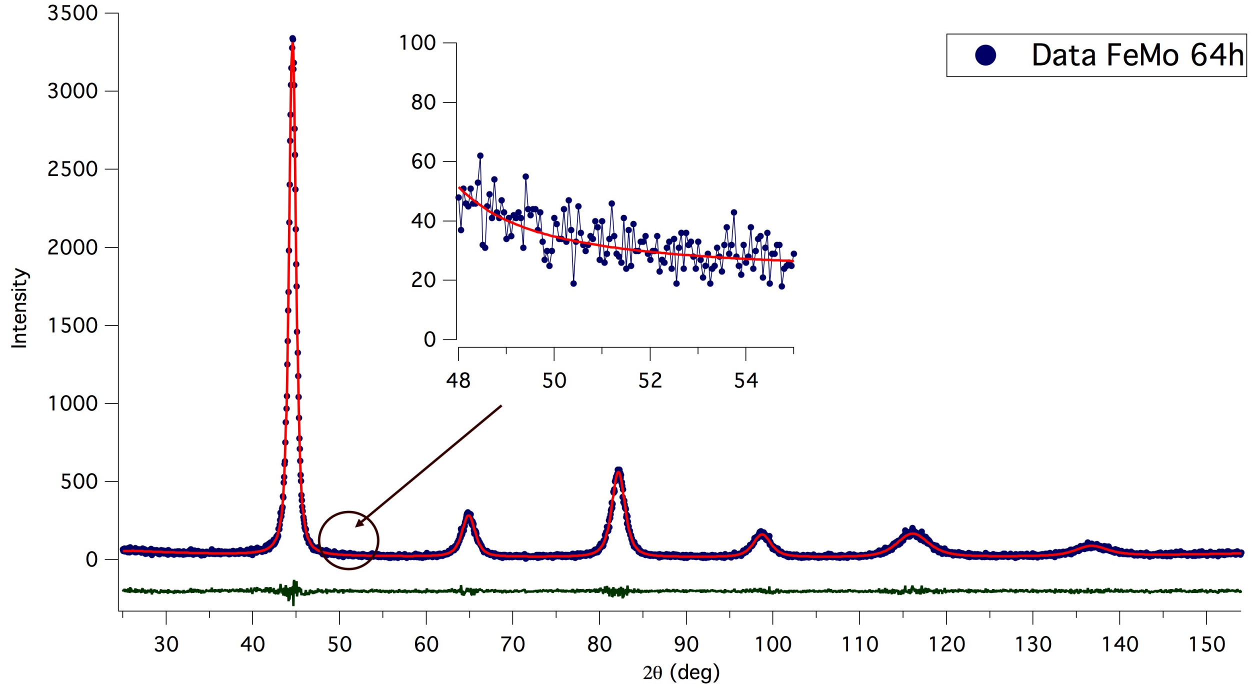

Figure SI3. XRD pattern of the FeMo powder of this study, ball milled for increasing time, from 8h (top) to 64 h (bottom). Experimental data (full symbol) are shown with the modelling result (see text of the main article for details); insets show the region with oxide peaks. Difference between experimental data and modelling (residual) is shown below. Up to 16h traces of oxide phases can be identified (arrow); these features disappear for 32h and longer milling times. Oxidation of powders ground for less than 32h takes place when jars are opened after the milling process, showing a typical pyrophoric behaviour. This behaviour is not observed for 32h and longer milling time, when the surface of agglomerate particles is passivated by the Cr-Ni contamination form jar and balls. This sequence also shows how the quality of the powder pattern modelling improves with the milling time.

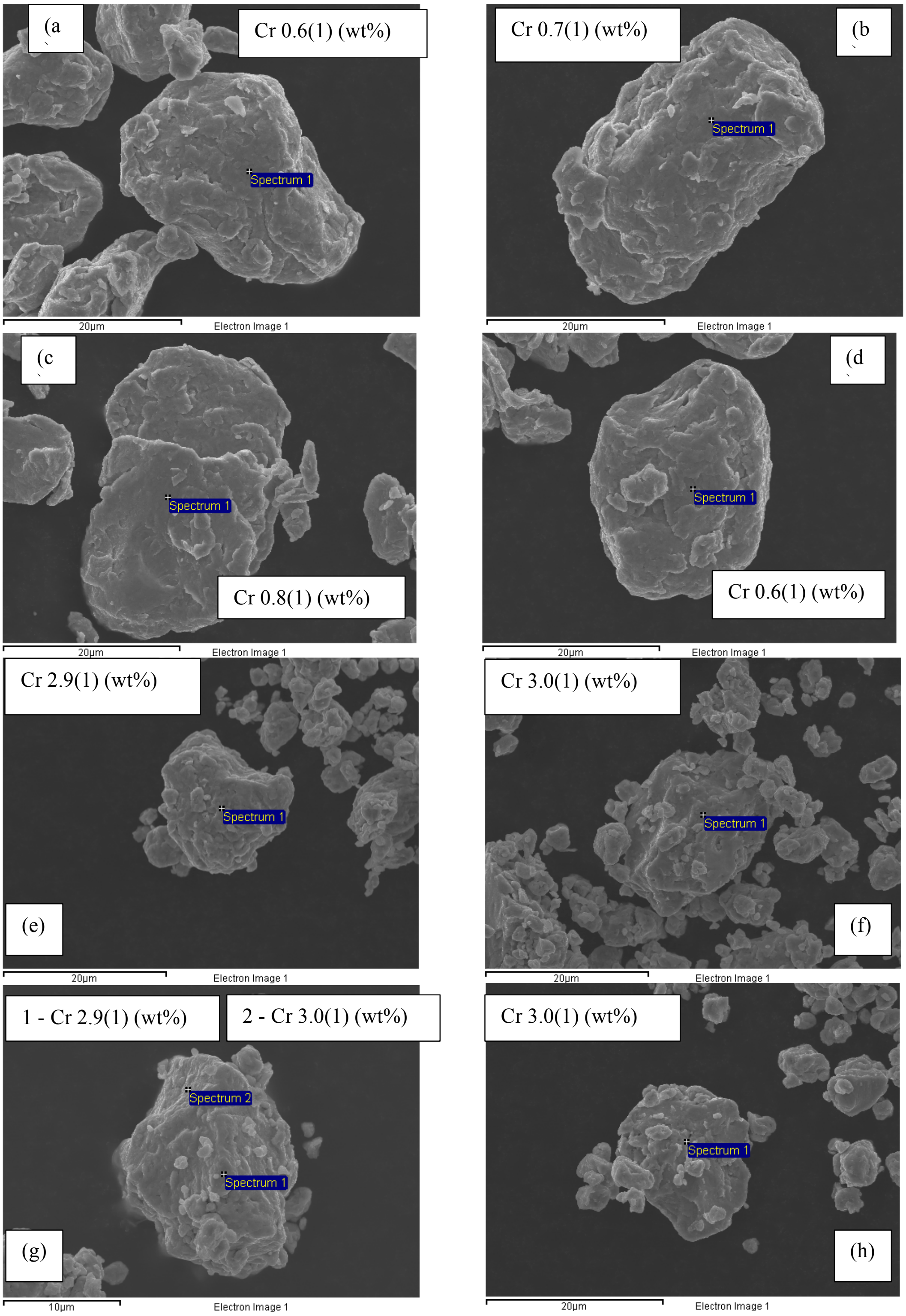

Figure SI4. SEM micrographs of the ball milled FeMo powder with indication of the Chromium content obtained by EDAX; (a,b,c,d) refer to a sample from a previous study, ball milled for 128 hours with similar grinding conditions[1]; (e,f,g,h) refer to the present study. The FeMo powder of pictures (a,b,c,d) proved to be stable over more than ten years, with no visible oxidation.[2] The more effective grinding conditions used in the present study give a higher Cr contamination which enhances the stability in time against oxidation. As shown in (g), Cr contamination is rather uniform in agglomerates.

2. SAXS measurement

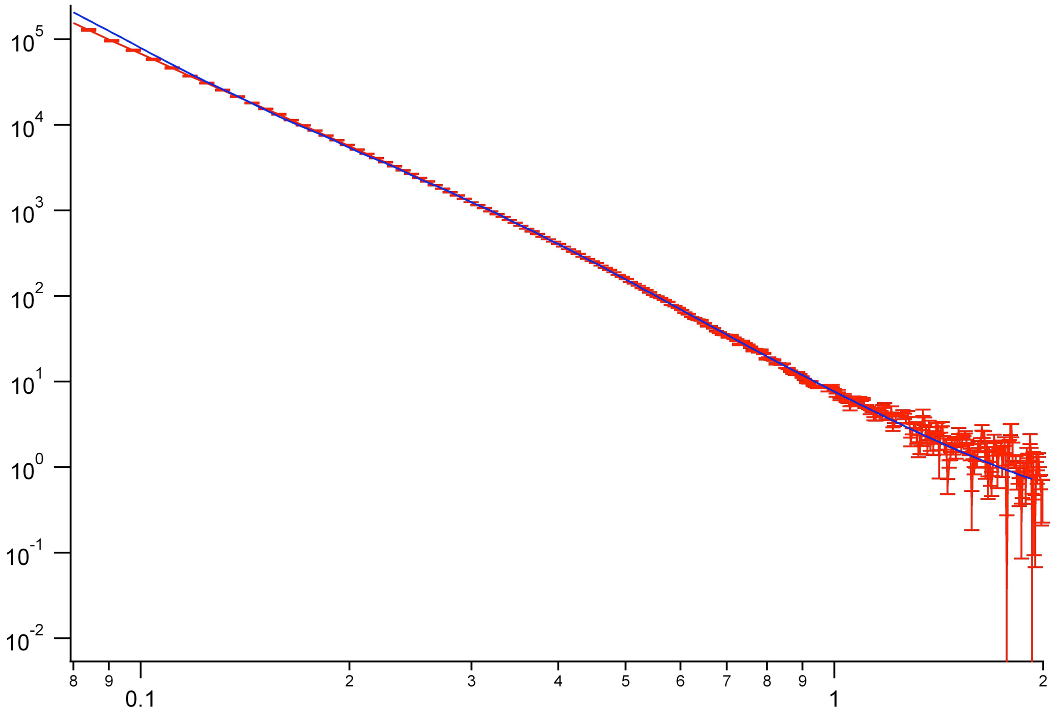

Figure SI4. SAXS pattern with fit of lognormal distributed spheres with sticky hard sphere interaction potential: mean diameter 16.2 nm, s.d. 7.6 nm.

References

1. D'Incau, M., Leoni, M. & Scardi, P. High-energy grinding of femo powders. *J. Mater. Res.* 22, 1744–1753 (2007). DOI:10.1557/JMR.2007.0224.

2. Troian, A., Rebuffi, L., Leoni, M. & Scardi, P. Toward a reference material for line profile analysis. *Powder Diffr.* 30, S47–S51 (2015). DOI:10.1017/S0885715614001298.

Appendix A: Root mean square strain (r.m.s. strain or "microstrain")

The Fourier Transform of the peak profile given by inhomogeneous strain can be written as:

$$A^D_{\{hkl\}} = \exp\left[-Q^2L^2\left\langle\varepsilon^2_{\{hkl\}}(L)\right\rangle/2\right] = \exp\left[-Q^2\left\langle\Delta L^2_{\{hkl\}}(L)\right\rangle/2\right] \quad (1)$$

where $Q = 4\pi\sin(\theta)/\lambda$ is the modulus of the wavevector transfer vector Q, and L is the Fourier length, i.e., the distance, projected along Q, between couples of scatterers in the crystalline domain, and $\left\langle\varepsilon^2_{\{hkl\}}(L)\right\rangle$ is the variance of the strain distribution.[1] This approach is quite general, even if we limit it here to cubic materials, so to refer to the $\{hkl\}$ family of atomic planes or equivalently to the $\langle hkl\rangle$ family of directions. Eq. (1) also introduces the mean square displacement, $\left\langle\Delta L^2_{\{hkl\}}(L)\right\rangle$, used in Warren's plot as $\sqrt{\left\langle\Delta L^2_{\{hkl\}}(L)\right\rangle}$ vs. L. (alternative notations include: $Z^2_L = L^2\varepsilon^2_L = \left\langle\Delta L^2(L)\right\rangle = \overline{\Delta L^2}$.[2–4]
The general expression Eq. (1) can be used in the Wilkens-Krivoglaz theory of diffraction line broadening caused by dislocations:[5]

$$\left\langle\varepsilon^2_{\{hkl\}}(L)\right\rangle_1 = \frac{\rho\overline{C}_{\{hkl\}}b^2}{4\pi}f^*\left(\frac{L}{R_e}\right) \quad (2)$$

with f^*, the so-called Wilkens' function (a slowly varying function of $\frac{L}{R_e}$),[5] and $\overline{C}_{\{hkl\}}$, the average contrast factor, which for cubic materials can be written as:

$$\overline{C}_{\{hkl\}} = A + BH^2 = A + B\frac{h^2k^2+k^2l^2+l^2h^2}{\left(h^2+k^2+l^2\right)^2} \quad (3)$$

A and B can be calculated for different dislocation types, slip systems and elastic media.[6] In a system of edge and screw dislocations it is convenient to introduce the fraction of edge dislocations, f_E, so that A and B can be written as linear combinations of values for pure edge and pure screw types:

$$A = A_Ef_E + A_S(1-f_E) \quad (4a)$$

$$B = B_Ef_E + B_S(1-f_E) \quad (4b)$$

For dislocations in the primary slip system of α-iron, $1/2\langle111\rangle\{110\}$, A_E = 0.265280; B_E = -0.355950; A_S = 0.307288; B_S = -0.819979.[7] The Burgers vector modulus, $b = \frac{\sqrt{3}a_0}{2}$, is about 0.2485 nm for α-i-iron.

Micro-strain effects of grain boundary and grain-grain interactions can be treated according to a model recently proposed by some of the authors,[8,9] which extends an expression originally proposed by Adler and Houska,[2] including strain anisotropy as:

$$\left\langle\varepsilon^2_{\{hkl\}}(L)\right\rangle_2 = \Gamma_{hkl}\left(\frac{a'}{L} + b'\right) \quad (5a)$$

with

$$\Gamma_{hkl} = c' + d'\frac{h^2k^2+k^2l^2+l^2h^2}{\left(h^2+k^2+l^2\right)^2} \quad (5b)$$

As shown by Popa,[10] Γ_{hkl} is the general expression of the invariant form for the Laue group of cubic phases, functionally identical with the average contrast factor of Eq. (3). However, differently from A and B in Eq. (3), which are calculated for dislocations, c' and d' (as well as a' and b'), are just free parameters, to be adapted (i.e., refined) to fit the observed peak profiles.
It is not straightforward to account for different strain contributions, like dislocations and grain boundaries, as the corresponding strain fields interact to some degree. To keep a reasonable level of complexity in the microstrain modelling, a fair approximation is to consider a convolution of the peak profile components related to Eq. (2) and (5a). The combined effect, in terms of Fourier Transform, is given by the product of the corresponding Fourier Transforms:

$$A^D_{\{hkl\}} = A^{D1}_{\{hkl\}}A^{D2}_{\{hkl\}}\cdots = \exp\left[-Q^2L^2\left(\left\langle\varepsilon^2_{\{hkl\}}(L)\right\rangle_1 + \left\langle\varepsilon^2_{\{hkl\}}(L)\right\rangle_2\right)/2\right] \quad (6)$$

The microstrain terms, $\left\langle\varepsilon^2_{\{hkl\}}(L)\right\rangle_1$ and $\left\langle\varepsilon^2_{\{hkl\}}(L)\right\rangle_2$, are then added in the exponent of Eq. (6). For example, in the

case of 1 Edge dislocation stable in G5, $\left\langle \varepsilon^2_{\{hkl\}}(L) \right\rangle = \left\langle \varepsilon^2_{\{hkl\}}(L) \right\rangle_{Free} + \left\langle \varepsilon^2_{\{hkl\}}(L) \right\rangle_{1EdgeStable}$, which means convolving the effect of grain boundary (and neighbouring grains) with that of a stable dislocation in G5.

References

1. Warren, B. E. *X-ray Diffraction*, 251–314 (New York: Addison-Wesley, 1969).
2. Adler, T. & Houska, C. R. Simplifications in the x-ray line-shape analysis. *J. Appl. Phys.* **50**, 3282–3287 (1979). DOI:10.1063/1.326368.
3. Warren, B. E. & Averbach, B. L. The effect of cold-work distortion on x-ray patterns. *J. Appl. Phys.* **21**, 595–599 (1950). DOI:10.1063/1.1699713.
4. Warren, B. E. & Averbach, B. L. The separation of stacking fault broadening in cold-worked metals. *J. Appl. Phys.* **23**, 1059–1059 (1952). DOI:10.1063/1.1702352.
5. Wilkens, M. *Fundamental Aspects of Dislocation Theory*, vol. II, 1195–1221 (Washington, DC: National Bureau of Standards, 1970).
6. Martinez-Garcia, J., Leoni, M. & Scardi, P. A general approach for determining the diffraction contrast factor of straight-line dislocations. *Acta Crystallogr. Sect. A* **65**, 109–119 (2009). DOI:10.1107/S010876730804186X.
7. D'Incau, M., Leoni, M. & Scardi, P. High-energy grinding of femo powders. *J. Mater. Res.* **22**, 1744–1753 (2007). DOI:10.1557/JMR.2007.0224.
8. Leonardi, A. & Scardi, P. Dislocation effects on the diffraction line profiles from nanocrystalline domains. *Metall. Mater. Trans. A* 1–11 (2015). Published online. DOI:10.1007/s11661-015-2863-y.
9. Scardi, P. *et al.* Anisotropic atom displacement in pd nanocubes resolved by molecular dynamics simulations supported by x-ray diffraction imaging. *Phys. Rev. B* **91**, 155414 (2015). DOI:10.1103/PhysRevB.91.155414.
10. Popa, N. C. The (*hkl*) Dependence of Diffraction-Line Broadening Caused by Strain and Size for all Laue Groups in Rietveld Refinement. *J. Appl. Crystallogr.* **31**, 176–180 (1998). DOI:10.1107/S0021889897009795.
